# Sarcoma Arising from the Chest Wall : A Case Report

**DOI:** 10.7759/cureus.1604

**Published:** 2017-08-24

**Authors:** Aisha Akhtar, Sana Shah, Abu Baker Sheikh, Adeel Nasrullah, Shujaul Haq, Haider Ghazanfar, Muneeba Rizwan

**Affiliations:** 1 Surgery, Texas Tech Health Sciences Center Lubbock; 2 Student, Aga Khan University Hospital, Karachi; 3 Department of Internal Medicine, Shifa International Hospital; 4 Internal Medicine, Newark Beth Israel Medical Center; 5 MBBS, Fatima Memorial Hospital

**Keywords:** chest wall, sarcoma, surgery

## Abstract

Chest wall contains a wide array of tissues ranging from soft tissues like skin and muscle to bone. A variety of sarcomas can present with a painful or painless mass, which often requires histological testing for diagnosis. Chest wall sarcomas are very rare entities which are often growing slow . A multidisciplinary team is necessary for the management of chest wall sarcomas. We present a case of a 30-year-old male with spindle cell sarcoma of the chest wall and he underwent wide local excision along with surgical reconstruction.

## Introduction

Sarcomas are rare malignant tumors that are mesenchymal in origin [1]. They constitute 1% of all malignancies in adults and out of this approximately 10-15 % have been reported to appear in the chest wall [2-3]. Primary soft-tissue sarcoma of the chest wall is a rare disease. The most common presentation is a slowly growing mass that is asymptomatic until it becomes large enough to compress or invade the surrounding structures. By the time they are diagnosed, most tumors are already large and advanced with an average size of 15 cm at the time of diagnosis [4]. Diagnosis is confirmed by a plain chest X-ray and a computed tomography scan. An open or Tru-cut biopsy is performed for planning the course of the treatment [5]. The general approach to the treatment includes wide surgical resection with tumor-free margins along with preservation of the major neurovascular structures [2, 5]. This is followed by skeletal reconstruction and soft tissue coverage [2]. In our report, we discuss the course of management of a patient who presented with a large chest wall sarcoma. Informed consent was obtained from the patient for this study.

## Case presentation

A 30-year-old male, known case of chronic kidney disease (CKD), on hemodialysis presented to the outpatient clinic with the complaint of a lump and feeling of heaviness on the left side of the chest for the past three months. According to the patient, the mass had progressively increased in size during this time, especially during the last month. There was no history of pain, trauma, weight loss, cough, dyspnea or fever.

His past medical history was positive for obstructive uropathy due to recurrent calculi which had eventually lead to CKD. He was on hemodialysis for the past three months. He had a surgical history of left pyelolithotomy in 2010.

On examination, he was a healthy male who was in no apparent distress with stable vital signs. He was pale and had a dialysis catheter in the right internal jugular vein. There was no evidence of jugular venous distension. The chest examination showed obvious chest wall asymmetry with a mass on left chest measuring 10 cm x 10 cm, extending from the left anterior to the lateral chest expanding from the sixth to the ninth rib. Mass was firm, non-tender, non-fluctuant and adherent to deeper structures. However, the overlying skin was normal. On respiratory examination, there was an equal bilateral air entry with normal vesicular breathing. There were no added breath sounds. Rest of the examination was unremarkable.

Laboratory exam was significant for hemoglobin of 8.9 g/dl with the normal total lymphocyte count of 5400/ul and elevated serum creatinine 4.6 mg/dl.

A chest X-ray was carried out which showed a soft tissue mass on the left side of the chest with normal lung fields and ribs.

The computed tomography (CT) scan of the chest revealed normal lung without any mass consolidation or effusion. A soft tissue density lesion was noted which was abutting the left sided lower ribs and pleura. There was no bony erosions or lymphadenopathy.

Given his history, physical examination and the scans, a Tru-cut biopsy was planned. It came out positive for pleomorphic spindle cell lesion with a few atypical mitotic cells and areas of necrosis. There was suspicion of malignancy. Immunohistochemistry showed anti smooth muscle antibody (ASMA) positive, desmin and S100 proteins were negative. This supported a diagnosis of sarcoma

**Figure 1:**
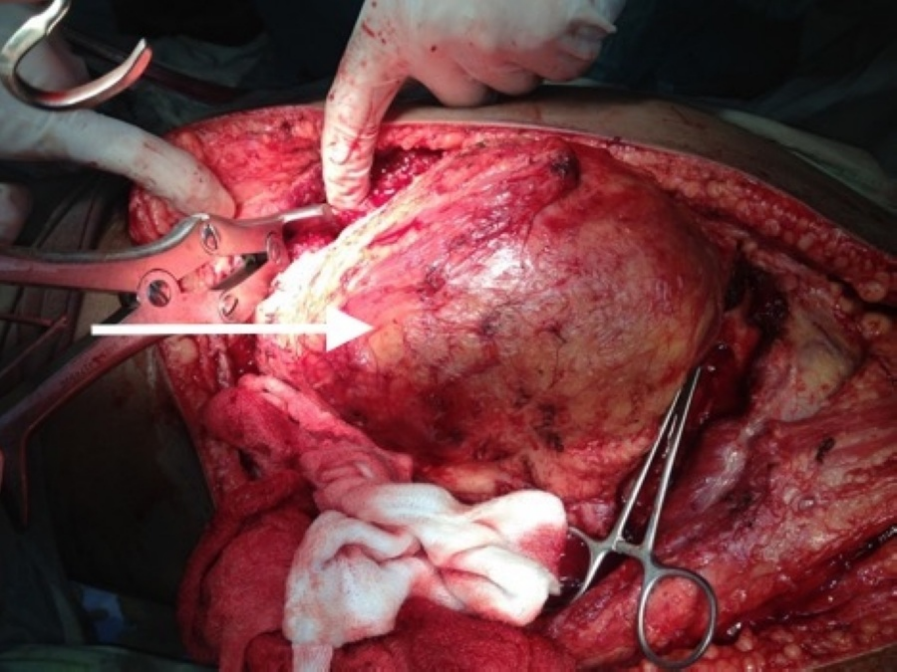
Intraoperative picture revealing well circumscribed left chest mass deeply adherent to underlying ribs.

His bone scan revealed mild increase uptake in left seventh and eighth ribs laterally, which was suspicious for early local bone invasion. This case was discussed in a multidisciplinary meeting of the hospital and surgery was recommended. The plastic surgery team was also consulted for reconstruction. Preoperative optimization was done and he underwent wide local excision along with surgical reconstruction. The mass was extending over his five lower ribs on the left, without any pleural involvement. This has been shown in Figure 1.

**Figure 2:**
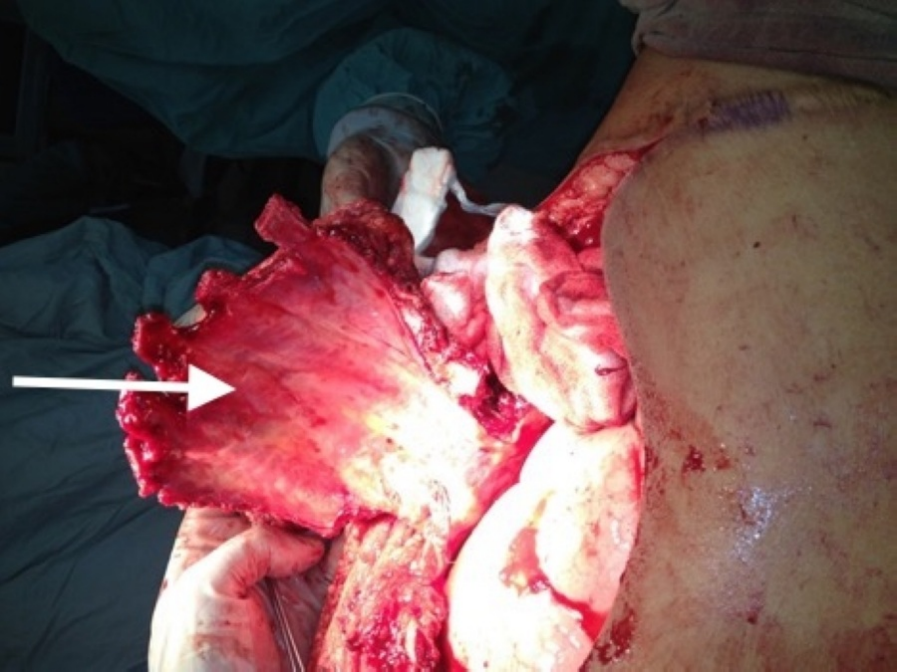
Intraoperative image revealed En-bloc excision of the left chest wall mass along with underlying ribs.

**Figure 3:**
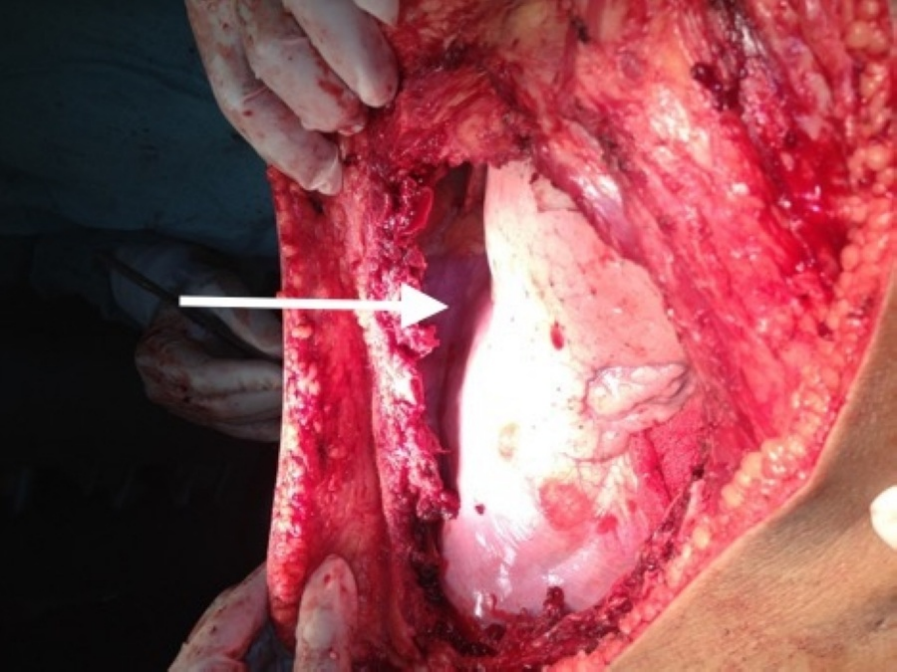
Intraoperative image showing defect after excision of the soft tissue mass.

The latissimus dorsi muscle was spared. En bloc excision of the mass with ribs was done and a wide margin was obtained. This has been shown Figure 2-3.

The plastic surgery team reconstructed his chest wall with a double Prolene® mesh (Ethicon US, LLC, Cincinnati, OH) for skeletal wall coverage and a latissimus dorsi flap was used to reconstruct soft tissue of the chest wall. The patient tolerated the procedure very well and was transferred to the intensive care unit on a ventilator. He was weaned off the ventilator successfully within 24 hours. The postoperative course was unremarkable and he was discharged home on the sixth postoperative day and had an uneventful recovery. The patient was advised regular follow-up to monitor his recovery and pulmonary function test. He was advised to follow-up in the oncology department every three to four months for the first three years following the operation and every six months for the following two years. After that yearly visit was advised. The chest X-ray was to be performed every six to 12 months for the first three years and then once a year to monitor any recurrence.

## Discussion

The chest wall tumors are rare and present as a clinical challenge for surgeons. Incorrect diagnosis, insufficient resection and ineffective reconstruction of large thoracic wall defects have resulted in high rates of perioperative morbidity and mortality. The extensive literature review and patient analysis with chest wall tumors have shown that surgical treatment may be the best treatment of choice for primary tumors and selected secondary tumors of the chest wall. Likewise, surgery has been proven to be the most successful curative option for patients with chest wall tumors [6]. Surgery with a wide margin is a safe and good technique for the treatment of primary chest wall tumors with acceptable morbidity and mortality [7].

In a study published by the Department of Surgical Oncology and Medical Statistics, Netherlands Cancer Institute, the five-year survival rate for wide local excision of primary sarcoma was 63%. The use of chemotherapy in chest wall sarcomas remains debatable. However, the use of radiation therapy in conjunction with resection has given a favorable response, especially in those cases which are resected with close or positive margins [2]. It also depends upon the stage of the disease, with the greatest benefit being attained in stage three and above [3]. Kachroo P, et al. reported 51 patients with primary chest wall sarcomas, underwent full-thickness resection. The results showed that local and distal recurrences were decreased by neoadjuvant systemic therapy and may improve survival in the patients [1]. The major factors that determine the prognosis include the histological grade, presence or absence of metastatic disease and attaining total resection [8]. 

Surgical resection of the tumor remains to be the treatment of choice [9]. The tumor has a tendency to infiltrate the ribs and surrounding structures. In most cases of malignant chest wall tumors, full thickness chest wall resection is needed. Therefore, thoracotomy along with resection of ribs and flap transfer to cover the skin defect is usually required. We followed a similar approach in this case as our patient underwent wide local excision along with surgical reconstruction and it led to an uneventful recovery.

## Conclusions

The chest wall sarcomas are an interesting and rare occurrence. Management should ideally involve a multidisciplinary approach, surgery being the mainstay of the treatment for a limited disease is often curative. Augmentation with radiotherapy and chemotherapy depends upon the extent of the disease. Timely diagnosis and management offer a favorable prognosis. Therefore, formulation of standard guidelines regarding the treatment of chest wall sarcomas is needed for proper management.
